# Phylogeographic Pattern of the Assassin Bug *Sycanus bifidus* Inferred from Mitochondrial Genomes and Nuclear Genes

**DOI:** 10.3390/biology13050305

**Published:** 2024-04-28

**Authors:** Suyi Chen, Zhenyong Du, Ping Zhao, Xuan Wang, Yunfei Wu, Hu Li, Wanzhi Cai

**Affiliations:** 1 Department of Entomology, MOA Key Lab of Pest Monitoring and Green Management, College of Plant Protection, China Agricultural University, Beijing 100193, China; chensuyiii@126.com (S.C.); caudzy@126.com (Z.D.); bs20213190971@cau.edu.cn (X.W.); 2 Sanya Institute of China Agricultural University, Sanya 572025, China; 3 Key Laboratory of Environment Change and Resources Use in Beibu Gulf (Ministry of Education) and Guangxi Key Laboratory of Earth Surface Processes and Intelligent Simulation, Nanning Normal University, Nanning 530001, China; zpyayjl@126.com; 4 Department of Plant Protection, Kaili University, Kaili 556000, China; 5 College of Biology and Food Engineering, Chuzhou University, Chuzhou 239000, China; wuyunfeiakx@126.com

**Keywords:** *Sycanus bifidus*, assassin bug, Pleistocene glaciation, phylogeography, sea-level change, climatic fluctuation

## Abstract

**Simple Summary:**

This study focuses on the assassin bug *Sycanus bifidus*, a widespread species in southern China, by analyzing its genetic data to trace how the Pleistocene’s climate and geography influenced its distribution and evolution. There are two main genetic groups, which suggests that past climatic conditions and sea-level rises, especially concerning Hainan Island, profoundly impacted the spread and distribution of these bugs. This study’s insights into the assassin bugs’ historical population dynamics and adaptations to environmental changes emphasize the role of historical climatic fluctuations in shaping the distribution of species.

**Abstract:**

The assassin bug *Sycanus bifidus* has a wide distribution across southern China. This study explored its distribution and evolution by analyzing mitochondrial and nuclear ribosomal RNA genes, revealing how Pleistocene climate and geological changes shaped its phylogeography. We identified two main clades, A and B, that diverged in the Middle Pleistocene. Hainan Island’s populations form a unique group within Clade A, suggesting that the Qiongzhou Strait served as a dispersal corridor during glaciation. Rising sea levels likely separated the Hainan population afterward. Ecological niche modeling showed that both populations have been viable since the last interglacial period, with demographic analyses indicating possible expansions during the Middle and Late Pleistocene, driven by favorable climates. This study highlights the significant effects of Pleistocene sea-level and climatic changes on the distribution and evolution of *S. bifidus* in China.

## 1. Introduction

Environmental and geological changes are pivotal in shaping the geographical distribution and genetic structure of organisms, as documented in numerous studies [1,2,3]. These changes can lead to rapid speciation, extinction, and demographic expansion or contraction across different taxa [1,4,5]. The Quaternary glaciation period, which was characterized by drastic climatic oscillations, profoundly affected the distribution patterns of organisms worldwide [6,7]. The glacial and interglacial cycles also induced geological changes, such as fluctuations in sea levels and the cyclical connection and disconnection of land bridges, which further impacted the dispersal and isolation of species [2,8]. Moreover, complicated topographies and localized climates have served as refugia for organisms during glaciations, facilitating post-glacier dispersal [5,9,10].

Hainan Island, situated in the transitional zone between tropical and temperate climates in the South China Sea, exemplifies the impact of these changes. The island, which is currently separated from Mainland China by the Qiongzhou Strait, was connected to the mainland until the strait’s formation due to volcanic activity and rising sea levels ~2.50–2.00 million years ago (Ma) [2,11,12]. The Pleistocene sea-level changes led to the repeated connection of Hainan Island with the mainland, with land bridges forming during specific periods during the Middle Pleistocene [2,13,14]. These geological events facilitated biotic exchanges between Hainan and neighboring regions, promoting genetic diversity and divergence [15,16]. Thus, island populations can exhibit significant intraspecific genetic divergence, local adaptation, and even signs of incipient speciation that can be largely attributed to climate-mediated sea-level changes [2,5,17].

**Figure 1:**
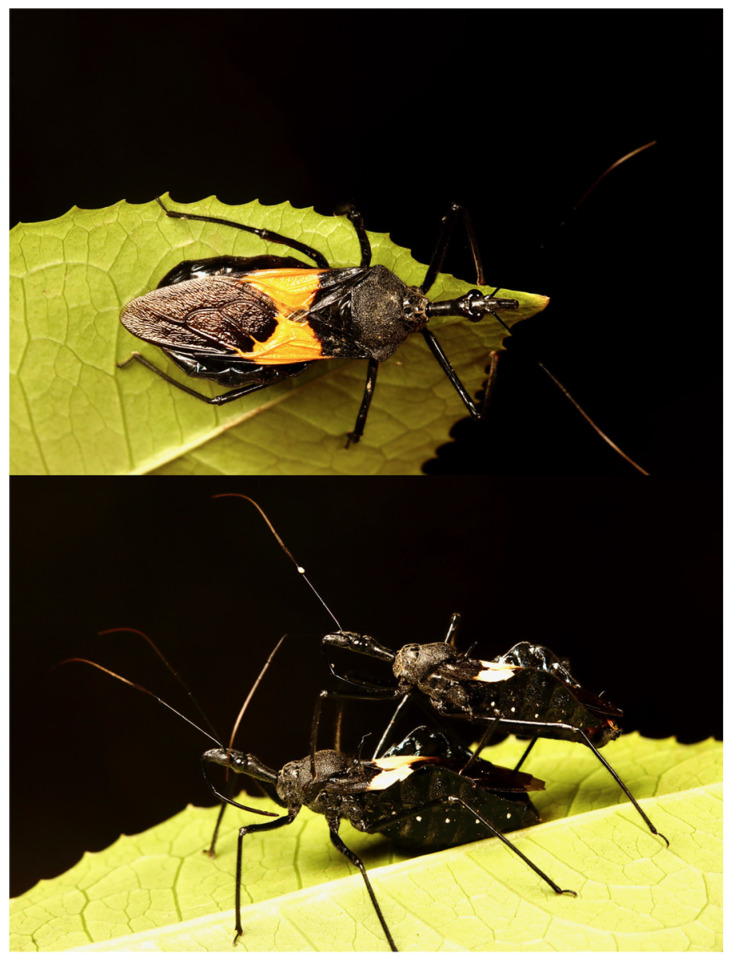
Morphological views of *Sycanus bifidus*. The upper image displays the dorsal view (top view), while the lower image presents the lateral view (side view).

The assassin bug *Sycanus bifidus* (Fabricius, 1787), a member of the Harpactorinae subfamily (Hemiptera: Reduviidae), was initially described in China [18,19]. It is synonymous with three other names: *S. croceovittatus* Dohrn, 1859; *S. leucomesus* Walker, 1873; and *S. villicus* Stål, 1863 [19]. The *Sycanus* genus is distinguished by several unique morphological features. For instance, its head is much longer than its pronotum, its scutellum is equipped with a spine or tubercle, and the connexivum of its abdomen is pronouncedly laterally dilated [19]. *S. bifidus* exhibits a striking coloration, with a black and slightly shiny body, a yellow or orange apical half corium of the hemelytron, and two round white spots at each sternum of the abdomen (Figure 1). As a polyphagous predator, *S. bifidus* has been observed to control a variety of pest species, highlighting its potential as a biological control agent in both forest and agricultural settings [20,21]. Despite its recognized role in pest management, the evolutionary history and biogeographical spread of *S. bifidus*, particularly its introduction and establishment on Hainan Island, remain largely unexplored. 

The development of molecular biology techniques has greatly facilitated the use of molecular markers in taxonomic identification and systematic studies [22,23,24]. Studies using different molecular markers have provided novel insights into the phylogeographic patterns and population genetic structures of different species. Mitochondrial genomes (mitogenomes) are particularly noted for their clear orthology, rapid evolutionary rate, and higher probability of displaying shorter coalescence times than nuclear loci, which establishes them as potent tools for filling the gap in evolutionary information provided by the nuclear genome [25,26]. Phylogeographic studies leveraging mitogenomic data have been instrumental in clarifying species’ divergent patterns and demographic histories [27,28,29,30]. However, the mitogenome’s ability to provide comprehensive evolutionary insight is limited, since it represents a single, non-recombining haplotype locus and only reflects a maternal inheritance pattern [26]. Multiple-copy nuclear ribosomal RNA genes have also been effectively sequenced as complementary markers to mitochondrial genes [31,32]. Nuclear internal transcribed spacer (ITS) regions, known for their high sequence variability, have proven successful for use in species differentiation [33]. Analyzing the phylogeographic pattern of the assassin bug *S. bifidus* will contribute to our understanding of its evolutionary history and support its application as a pest control agent [5,34,35].

This study employed an integrative approach by combining data from the complete mitogenome and nuclear ribosomal RNA genes to elucidate the phylogeographic patterns and demographic histories of *S. bifidus* in southern China. Our study aimed to achieve the following objectives: (1) delineate the phylogeographic structure of *S. bifidus* populations across southern China, (2) ascertain the process by which *S. bifidus* migrated to Hainan Island from Mainland China across the Qiongzhou Strait, and (3) examine the effects of Pleistocene climatic fluctuations and geological events on the phylogeographic structure of *S. bifidus*. These objectives were designed to provide a comprehensive understanding of the evolutionary dynamics and biogeographical distribution of *S. bifidus*, which will facilitate efforts in conserving the diversity of this species.

## 2. Materials and Methods

### 2.1. Sampling and DNA Extraction

**Figure 2:**
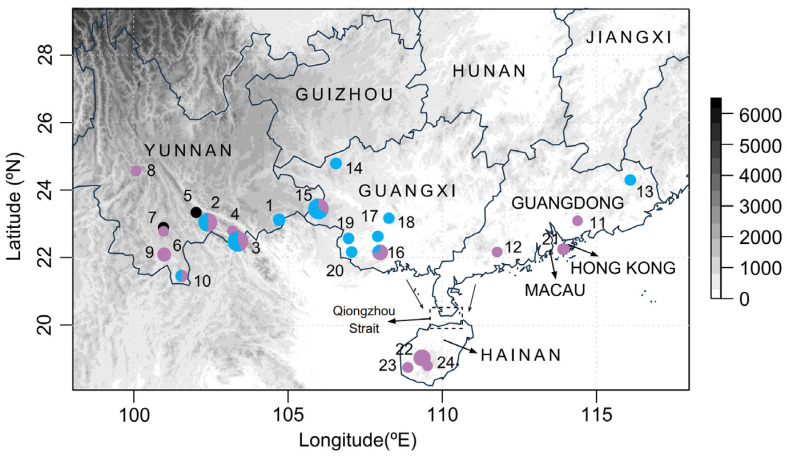
Sampling localities and genetic clustering of *S. bifidus*. This map shows the distribution of sampling sites across the study area, with pie charts indicating the proportion of BAPS clusters at each locality. The circle colors correspond to the two identified BAPS clusters, and the black circles denote the samples that were not included in the BAPS analysis due to incomplete nuclear rRNA genes. The pie chart sizes are proportional to the number of samples collected at each site. Detailed locality information is provided in Table S1.

A total of 65 specimens were collected from 24 locations in southern Mainland China and Hainan Island and were stored in absolute alcohol at −80 °C before DNA extraction (Figure 2 and Table S1). The total genomic DNA was extracted from the muscles of all specimens using a DNeasy Blood and Tissue Kit (QIAGEN, Hilden, Germany) by following the manufacturer’s instructions.

### 2.2. Mitogenome and Nuclear rRNA Gene Sequencing

Next-generation sequencing libraries were constructed with an average insert size of 350 bp. All the libraries were sequenced to acquire at least 6 Gb of raw reads in the 150 bp paired-end mode on an Illumina NovaSeq 6000 platform (San Diego, CA, USA) at Berry Genomics (Beijing, China). The raw reads were trimmed from adapters using Trimmomatic [36]. De novo assemblies for each sample were generated using IDBA-UD [37], iterating the value of *k* from *k*min = 41 to *k*max = 141, with a minimum contig length of 1500 bp and a similarity of 98%. We collected mitogenomes and nuclear rRNA genes to better understand the phylogeographic patterns of *S. bifidus*. For the mitogenomes, BLAST searches with a minimum of 96% similarity were performed to select the best-fit mitogenomic scaffolds from the assembled scaffolds, using the published mitogenome of *S. bifidus* (GenBank accession MT535605) as a bait sequence. The newly obtained mitogenome sequences were annotated using MitoZ [38]. For the nuclear rRNA genes, BLAST searches with at least 92% similarity were conducted to obtain the best-fit scaffold from the assembled scaffolds, using the published sequences of the 18S ribosomal RNA (rRNA), internal transcribed spacer 1 (ITS1), 5.8S rRNA, ITS2, and 28S rRNA of the assassin bug *Brontostoma colossus* (Hemiptera: Reduviidae) (GenBank accession KM278219) as bait sequences. The gene boundaries for the nuclear rRNA genes were manually annotated based on their alignment with *B. colossus*’s sequence. We obtained a complete mitogenome for all 65 samples and nuclear rRNA gene sequences for 59 samples. *Sycanus croceus* and *Sycanus falleni* were designated as outgroups for the phylogenetic analyses. All sequences of *S. bifidus* and the outgroup species generated in this study were deposited in GenBank under the accession numbers OR734017–OR734077 and OR743562–OR743622.

### 2.3. Genetic Diversity and Population Differentiation

Thirteen protein-coding genes (PCGs) of the mitogenome were individually aligned using the TranslatorX online platform (http://www.translatorx.co.uk, accessed on 16 February 2024) [39] based on codon-based multiple alignments with all the stop codons removed. All the other mitochondrial genes, including 22 transfer RNAs (tRNAs) and two rRNAs, and the nuclear rRNA genes were aligned using MAFFT 7.0, an online server with the G-INS-i strategy [40]. The 37 mitochondrial genes were analyzed both separately and in conjunction with the nuclear rRNA genes.

To assess how the genetic diversity varied across geographic populations, the number of polymorphic sites (*S*), the number of haplotypes (*H*), the haplotype diversity (*Hd*), and the nucleotide diversity (*π*) were calculated to estimate the DNA polymorphism for the mitochondrial and nuclear rRNA genes using DnaSP 6.0 [41]. BAPS 6.0 [42] was used to perform a Bayesian analysis of the population structure based on a concatenated dataset of mitochondrial and nuclear rRNA genes under the spatial clustering of groups of individuals.

### 2.4. Phylogenetic Relationship and Haplotype Network Reconstruction

Phylogenetic analyses were conducted to investigate the phylogeographic patterns within *S. bifidus*. To ensure the reliability of our phylogenetic analyses, we employed two distinct methods: the concatenation method and the coalescent method. In the concatenation method, phylogenetic trees were constructed based on a concatenated dataset of mitochondrial and nuclear rRNA genes and another dataset that only included mitochondrial genes. The maximum-likelihood (ML) method was conducted in IQ-TREE 1.6.5 [43], which implemented 1000 replicates with the ultrafast bootstrap approximation approach for bootstrap support [44]. Best-fit schemes and substitution models (shown in Table S2) were selected before the phylogenetic analyses using PartitionFinder 2 [45] based on the “greedy” algorithm and Akaike information criterion (AIC). Additionally, Bayesian inference (BI) analyses were performed, involving two simultaneous Markov chain Monte Carlo (MCMC) runs of two million generations, with the trees sampled every 1000 generations. The first 25% of the trees in each MCMC run were discarded as burn-ins. The convergence was ensured with an average standard deviation of split frequencies (ASDSF) below 0.05. For the coalescent methods, individual gene trees for each mitochondrial and nuclear rRNA gene were constructed using IQ-TREE with the “automatically ModelFinder searching” mode and 1000 ultrafast bootstraps [46]. The multispecies coalescent (MSC) model, which addressed incomplete lineage sorting, was run by ASTRAL-III v5.6.1 [47]. Local branch supports were estimated from quartet frequencies using the ASTRAL script (https://github.com/xtmtd/Phylogenomics/tree/main/scripts, accessed on 16 February 2024). 

Haplotype network analyses were conducted to explore the nested relationships among different haplotypes within *S. bifidus*. The haplotype data were generated using DnaSP. Median-joining networks [48] of haplotypes were constructed for the concatenated dataset of mitochondrial and rRNA genes, and for mitochondrial genes alone, using PopART [49]. The haplotype frequency was obtained using Arlequin 3.5 [50].

### 2.5. Estimation of Divergence Time

We estimated the divergence time of the main phylogeographic clades of *S. bifidus* in southern Mainland China and Hainan Island using BEAST 2.4.8 [51]. We used a molecular clock to estimate the divergence time because no fossils were available for the calibration of this species. Specifically, the mitochondrial cytochrome c oxidase subunit I gene (*COX1*) was analyzed as a separate partition with a fixed substitution rate of 1.77% per site per million years [52]. The other protein-coding genes, tRNA genes, mitochondrial rRNA genes, and nuclear rRNA genes were evaluated in separate partitions, with the substitution rates estimated accordingly. For each partition, the best-fit model of GTR + I + G was determined using PartitionFinder 2 based on the “greedy” algorithm and AIC (Table S3). The relaxed clock log normal model was applied with the birth–death model for the tree prior, and with linked tree and clock models and unlinked site models. Two independent MCMC runs of 100 million generations were performed, with trees sampled every 1000 generations in BEAST on CIPRES Science Gateway V 3.3 [53] (http://www.phylo.org, accessed on 22 February 2024). Tracer 1.7 [54] was used to verify whether the MCMC runs had reached a stationary distribution based on the effective sample size of each estimated parameter, where we required an effective sample size > 200 for the posterior, prior, and tree likelihoods. The LogCombiner and TreeAnnotator programs in the BEAST software were used to combine the resulting trees for each replicate and calculate the consensus tree with the trees sampled every 10,000 generations. The divergence times were annotated with the “mean height” after discarding the first 25% trees as burn-in with the posterior probability limited to 0.5.

### 2.6. Demographic History Inference

The neutral tests of Tajima’s *D* [55] and Fu’s *Fs* [56] were performed using the Arlequin software with default parameters based on the mitochondrial genes alone. The *p*-values of these parameters were obtained by comparing the observed statistical values with 1000 simulated values under the hypotheses of selective neutrality and population equilibrium [50]. A mismatch distribution analysis of the significance of the sum of the squared deviations (*SSD*) and Harpending’s raggedness index (*r*) was performed based on the mitochondrial genes alone using the Arlequin software with 1000 bootstrap replicates to detect the expansion through the linear fitting of the observed and simulated curves [50]. Bayesian skyline plots (BSPs) were obtained for all the samples as a whole, two phylogeographic clades, and the Hainan Island population using the coalescent Bayesian skyline model for the prior tree in the BEAST2 software, based on mitochondrial and nuclear rRNA genes. The same partitions and substitution rates were applied, consistently with those in the estimation of divergence time, and the obtained results of the divergence time were applied to calibrate the most recent common ancestor (MRCA) in each corresponding BSP analysis. Two independent MCMC runs of 100 million generations were performed, with trees sampled every 1000 generations as well. We determined the convergence and the effective population size changes over time using Tracer, discarding the initial 10% of generations as burn-in.

### 2.7. Ecological Niche Modeling

Species distribution modeling was used to predict the potential and suitable distribution areas of the species. We compared the present and predicted areas for the last glacial maximum (LGM; ~22 thousand years ago, Ka) and the last interglacial (LIG; 120–140 Ka) periods. Information on the sample sites in the present study was used as the distribution data (Table S1). The model of interdisciplinary research on climate (MIROC) was used in the LGM and LIG predictions. Climate data for the present (2.5 min resolution), LGM (2.5 min resolution), and LIG (30 arc-second resolution) periods were obtained from the WorldClim database (https://www.worldclim.com/version1, accessed on 27 September 2023) [57]. The climate layers were generated in ArcGIS 10.0 (https://www.esri.com/en-us/arcgis, accessed on 17 October 2023). To avoid overfitting the model, we filtered the initial variables based on the results of Pearson’s correlation tests [58] using IBM SPSS Statistics 21.0 (Chicago, IL, USA). Highly correlated variables (|*r*|  ≥  0.85) were removed, leaving 10 variables (BIO1, annual mean temperature; BIO5, max temperature of warmest month; BIO6, min temperature of coldest month; BIO8, mean temperature of wettest quarter; BIO9, mean temperature of driest quarter; BIO10, mean temperature of warmest quarter, BIO11, mean temperature of coldest quarter; BIO14, precipitation of driest month; BIO17, precipitation of driest quarter; BIO19, precipitation of coldest quarter) for the analysis.

The suitable distribution area of *S. bifidus* was predicted using Maxent 3.3.3k [59], with 75% of the species records used for training and 25% used to test the model. The parameters used in Maxent were as follows: maximum number of background points = 10,000, regularization multiplier = 1, replicates = 10, replicated run type selected subsample, maximum iterations = 5000, convergence threshold = 10^−5^, and applied threshold rule = 10 percentile training presence. The model performance was evaluated according to the area under the curve (AUC) of the receiver-operating characteristic plot. The index of suitability ranged from 0 to 1 and the results of the predicted distributions on the grid maps were visualized using ArcGIS.

## 3. Results

### 3.1. Variations in Mitogenomes and Nuclear rRNA Genes

A total of 65 whole mitogenome sequences with 37 mitochondrial genes, including 13 protein-coding genes, 22 tRNA genes, and 2 rRNA genes, and a control region were obtained from 24 geographical populations (Tables S1 and S4). The length of the sequences varied from 15,640 to 15,647 bp, with 28.2–28.3% GC content. No large variations in gene arrangement or non-coding regions were detected among the different populations. Based on the mitogenomic dataset, including all PCGs, tRNA, and rRNA genes (14,584 bp), 62 haplotypes were detected with an overall haplotype diversity (*Hd*) of 0.998, segregating site (*S*) of 1027, and nucleotide diversity (*π*) of 0.00714.

For the nuclear rRNA dataset, 59 sequences were obtained from 22 geographical populations, with complete 18S-ITS1-5.8S-ITS2-28S sequences (Tables S1 and S4). The length of the sequences varied from 6844 to 6848 bp, with 44.5% GC content. Among all the populations, only four haplotypes were identified, with a haplotype diversity of 0.192 and a nucleotide diversity of 0.00005.

For the analysis on concatenated mitochondrial and nuclear rRNA datasets, 56 haplotypes were observed, comprising 21,435 bp from 59 individuals in 22 collection locations (Figure 2 and Table S1). The average haplotype diversity for all individuals was 0.998 and the average nucleotide diversity was 0.00490 (Table S4).

### 3.2. Phylogeographic Pattern

**Figure 3:**
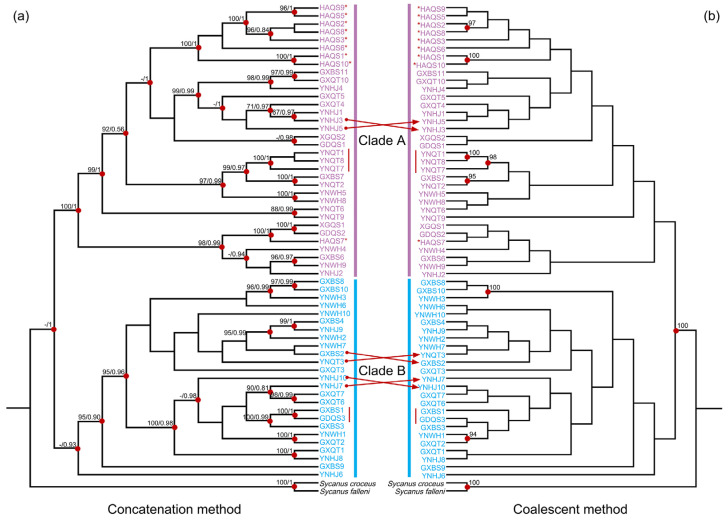
Phylogenetic relationships of *S. bifidus*, derived from mitochondrial and nuclear rRNA gene sequences. (**a**) Tree topology conducted with the concatenation method. Bootstrap support values from maximum-likelihood analysis with IQ-TREE and posterior probabilities from Bayesian inference analysis with MrBayes are represented by numbers at the nodes. Red dots indicate high support (bootstrap support > 90; posterior probability > 0.9). (**b**) Tree topology from multispecies coalescent (MSC) analysis performed with ASTRAL. Numbers at the nodes show posterior probabilities (MSC), with red dots highlighting high support (posterior probabilities > 90%). (**a**,**b**) Phylogeographic clades are differentiated by colors. Individuals from Hainan Island are denoted with red asterisks, and those sharing the same haplotype are indicated with red lines. Topological discrepancies between concatenation and coalescent methods are indicated with red arrows.

The BAPS analysis based on the concatenated dataset of mitochondrial and nuclear rRNA genes classified all samples into two clusters (Figure 2). One cluster included partial haplotypes from the Yunnan, Guangxi, and Guangdong regions. The other cluster included the remaining haplotypes as well as those from Hong Kong and Hainan Island (Figure 2). The phylogenetic analyses employing both concatenation and coalescent methods corroborated the BAPS findings, delineating two major phylogeographic clades (Clades A and B) that aligned with the two clusters in the BAPS results (Figure 3). This phylogeographic structure was maintained even when only mitochondrial genes were analyzed (Figure S1). Upon closer examination of the genetic diversity within these clades, Clade A (*π* = 0.00477) had a higher diversity than Clade B (*π* = 0.00445) (Table 1). Interestingly, all but one individual from Hainan Island (marked with red asterisks in Figure 3) formed a distinct and monophyletic group in the phylogenetic trees. To understand the divergence and origin of the Hainan Island group, we analyzed the Hainan Island population separately. The Hainan Island population (*N* = 10, *S* = 181, *H* = 10, *Hd* = 1.000, and *π* = 0.00307) exhibited a lower nucleotide diversity than the two main clades.

### 3.3. Divergence Time

**Figure 4:**
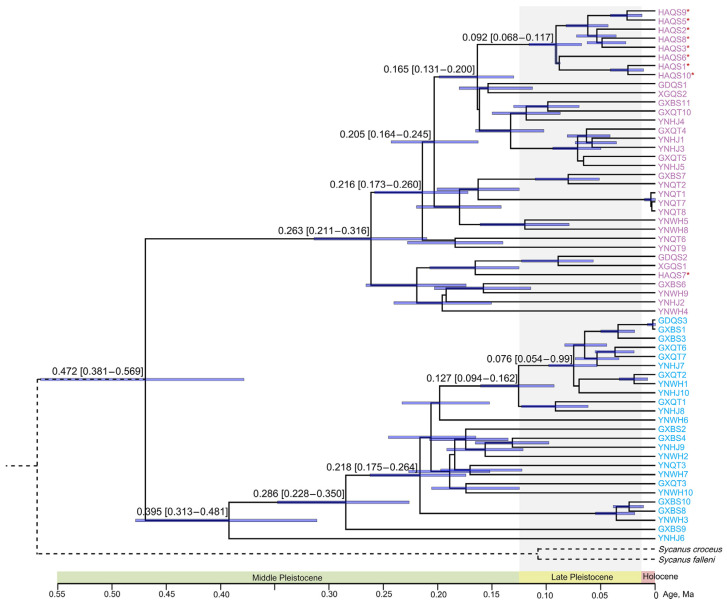
Divergence time estimation for *S. bifidus* using both mitochondrial and nuclear rRNA genes. Taxa labels represent individual specimens analyzed. Node mean age estimates are provided with their respective 95% highest posterior density (HPD) intervals, denoted by purple bars. Distinct phylogeographic clades are color-coded. Individuals from Hainan Island are identified with a red asterisk.

The BEAST method was utilized to estimate the divergence times of *S. bifidus*, providing crucial insights into the evolutionary history of this species (Figure 4). The estimation of the divergence time suggested that the MRCA age of *S. bifidus* was 472 Ka (95% HPD intervals: 381–569 Ka). The MRCA ages of Clades A and B were 263 Ka (HPD interval: 211–316 Ka) and 395 Ka (HPD interval: 313–481 Ka), respectively, supporting Middle Pleistocene Quaternary divergence. All the Hainan Island individuals except one diverged within Clade A at 165 Ka (HPD interval: 131–200 Ka). The MRCA age of the Hainan Island group was 92 Ka (HPD interval: 68–117 Ka) during the Late Pleistocene Quaternary.

### 3.4. Haplotype Network

**Figure 5:**
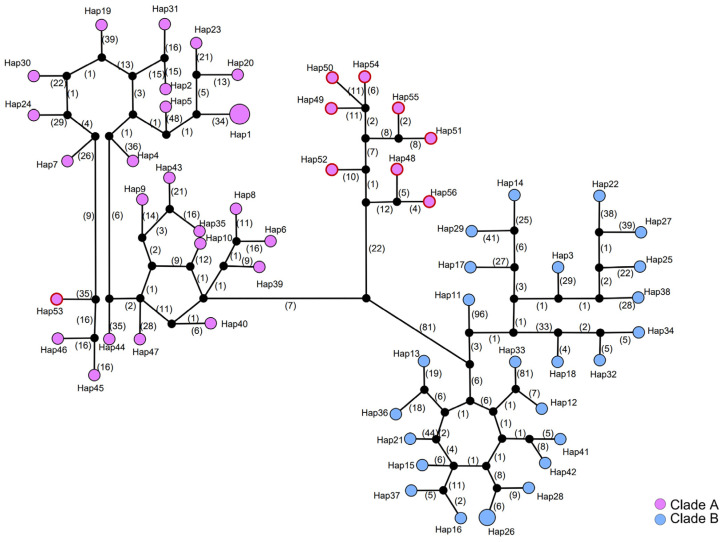
Haplotype network for *S. bifidus*, constructed from concatenated mitochondrial and nuclear rRNA genes. Each colored circle corresponds to a distinct observed haplotype, while black circles indicate hypothesized haplotypes not detected in the samples. Numbers along the connecting lines denote the number of mutation steps between haplotypes. Circle size is proportional to haplotype frequency. The colors of the circles match those used in Figure 3 to denote the same phylogeographic clade. Haplotypes identified in the Hainan Island population are enclosed in a red outline.

A haplotype network was constructed for the concatenated dataset of mitochondrial and nuclear rRNA genes (Figure 5 and Table S1), and for mitochondrial genes alone (Figure S2 and Table S1). Consistent with our observations of the population genetic diversity, we found a high level of haplotype diversity in both Clade A and Clade B. A total of 56 haplotypes were detected in 59 individuals, of which 54 were unique and represented by a single sample locality (Figure 5 and Table S1). Shared haplotypes were found only within the same clade. Most individuals from Hainan Island were closely clustered, typically connecting with another haplotype within 20 mutation steps. The closet connection between haplotypes from this Hainan Island cluster and other haplotypes in Clade A was between Hap52 and Hap40, which were separated by 46 mutation steps, and between Hap52 and Hap39, which were separated by 49 mutation steps. These two haplotypes from the southern Guangxi region act as genetic waypoints that hint at the potential routes of gene flow or historical migration between southern Mainland China and Hainan Island (Figure 5).

### 3.5. Demographic History

**Figure 6:**
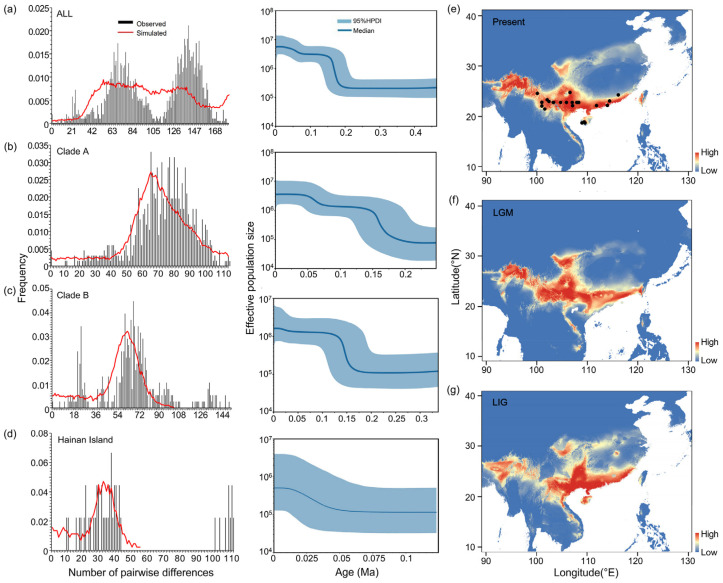
Demographic and distributional history of *S. bifidus*. Panels (**a**–**d**) illustrate mismatch distributions and Bayesian skyline plots (BSPs) for the mitogenome genes and nuclear rRNA datasets: (**a**) all samples, (**b**) Clade A, (**c**) Clade B, and (**d**) the Hainan Island population. Mismatch distributions are shown with vertical bars, while the expected distributions under a model of population expansion are overlaid in red. BSPs display the median effective population sizes over time (solid lines) within the 95% highest posterior density (HPD) intervals (shaded areas). Panels (**e**–**g**) depict hindcasted ecological niche models for East Asia during the current period (**e**), the last glacial maximum (LGM) (**f**), and the last interglacial (LIG) (**g**). Black circles indicate sampling localities contributing data to the model. Suitability ranges from low (blue) to high (red), with the index scaling from 0 to 1.

The application of neutral tests (*Tajima’s D* and *Fu’s Fs*), a mismatch distribution analysis, and BSP analyses collectively pointed towards a history of population expansion across the studied clades and the Hainan Island population. The negative and statistically significant values of *Tajima’s D* for all samples, Clades A and B, and the Hainan Island population suggested an excess of low-frequency polymorphisms, which is indicative of population expansion (Table 1). This was corroborated by *Fu’s Fs* test, which also showed negative values across these groups, although statistical significance was only reached for the all-sample set (Table 1). In the mismatch distribution analysis, the non-significant differences between the observed and simulated mismatch curves (based on both the *SSD* and the *r*) for all the groups suggested that the populations underwent expansion (Table 1 and Figure 6). The BSP analysis provided a temporal context for these demographic events, revealing two significant increases in the effective population size. For all the samples, and specifically for Clade A, these increases occurred around 160 Ka and 60 Ka, corresponding to the Middle and Late Pleistocene, respectively. Clade B’s expansion was noted to have begun around 150 Ka. The Hainan Island population showed a pattern of continuous and steady expansion, starting from approximately 50 Ka and aligning with the Late Pleistocene (Figure 6).

### 3.6. Ecological Niche Modeling

Ecological niche modeling (ENM) analyses for *S. bifidus* have provided robust model predictions, as evidenced by high AUC values for the present (0.994), last glacial maximum (LGM) (0.993), and last interglacial (LIG) (0.986) periods. These AUC values indicate excellent model performance and reliability in predicting the species’ suitable habitats. Among the climatic variables influencing the distribution of *S. bifidus*, the mean temperature of the coldest quarter (BIO11) emerged as the most significant contributor to the model predictions for the current and LGM periods, whereas the minimum temperature of the coldest month (BIO6) was most influential during the LIG period (Table S5). The mean temperature of the warmest quarter (BIO10) had the least impact across all models (Table S5). This suggested that temperature variables, particularly those associated with the coldest times of the year, played a critical role in determining the suitable habitats for *S. bifidus*. The ENM’s current predictions aligned well with the observed distributions of *S. bifidus*, confirming the suitability of southern coastal and southwestern China, as well as Hainan Island, as habitats for this species (Figure 6e). The comparison between the present and the LGM periods suggested a slight expansion of suitable areas in southern coastal China during the LGM, possibly due to the different climatic conditions prevailing during that period (Figure 6f). Conversely, during the LIG period, suitable habitats contracted, especially in southwestern China, though the suitability of southern coastal China and Hainan Island remained high (Figure 6g).

## 4. Discussion

### 4.1. Phylogeographic Pattern of S. bifidus in Southern China

Our study of the assassin bug *S. bifidus* in southern China uncovered significant insights into its phylogeographic structure and evolutionary history. This study delineated two primary phylogeographic clades within the *S. bifidus* populations in southern Mainland China and Hainan Island. An interesting observation from our study is the contrasting genetic diversity within these clades: Clade A exhibited a lower haplotype diversity, but a higher nucleotide diversity compared to Clade B. This pattern suggests that the accumulation of genetic diversity in these clades may be a result of their long evolutionary histories combined with restricted gene flow between populations [60,61]. The Hainan Island population displayed a relatively lower genetic variation, which could be attributed to a founder effect and subsequent genetic drift, or a smaller sample size. Founder effects occur when new populations are established by a small number of individuals from a larger population, leading to reduced genetic diversity in the new population compared to the source population [62,63,64,65,66].

### 4.2. Dispersal of S. bifidus from Mainland China to Hainan Island

The dispersal of *S. bifidus* from Mainland China to Hainan Island is a fascinating example of how geological events shape the biogeography of species. The formation of the land bridge of the Qiongzhou Strait during the Middle Pleistocene at three key intervals—0.80–0.60 Ma, 0.48–0.42 Ma, and 0.30–0.13 Ma—provided critical opportunities for the migration of terrestrial organisms between Mainland China and Hainan Island [2,13,14]. The divergence time for most individuals of *S. bifidus* on Hainan Island, estimated at 165 Ka, aligns with the period of the last formation of this land bridge, suggesting that the species might have utilized this natural corridor for dispersal during that time [67]. 

The haplotype network analysis showed closer connections between most Hainan Island haplotypes and certain haplotypes from southern Guangxi, indicating possible routes of migration across the Qiongzhou Strait. The observed genetic patterns, characterized by missing haplotypes and large mutation steps across the strait, indicate a prolonged divergence and restricted gene flow between the populations on either side of the Qiongzhou Strait. This suggests that the strait functioned as both a bridge and a barrier at different times, influencing the genetic structure of the local fauna.

The role of the Qiongzhou Strait as a dispersal corridor is not unique to *S. bifidus*. Similar biogeographic patterns have been documented in other species, such as the intermediate horseshoe bat (*Rhinolophus affinis*) [68] and Reeves’s butterfly lizard (*Leiolepis reevesii*) [69], underscoring the strait’s significant impact on species dispersal and speciation. While some studies have shown that the strait did not significantly hinder gene flow for certain species such as the oriental garden lizard (*Calotes versicolor*) [2], it is clear that its existence and the historical fluctuations in sea levels have played a pivotal role in shaping the evolutionary trajectories of many terrestrial organisms in this region.

### 4.3. Effects of Climatic Fluctuations on the Distribution Patterns of S. bifidus

The impact of Quaternary climatic oscillations and geological events has profoundly influenced the distribution and genetic diversity of extant assassin bug species, including *S. bifidus*. These climatic fluctuations, characterized by cycles of glaciation and interglaciation, led to the contraction of species’ ranges into refugial areas during cold periods and their expansion during warmer interglacial periods. Glacial refugia have been recognized for their role in preserving intraspecific diversity, serving as biodiversity hotspots and sanctuaries for species survival and evolution [1,9,70]. A previous study on the spinous assassin bugs *Sclomina* revealed that climatic fluctuations during the Pleistocene led to habitat fragmentation and the post-glacial expansion of this genus in East Asia, fostering allopatric speciation and intraspecific diversification within the genus [5].

The southwestern plateau of China, as a recognized biodiversity hotspot, acted as a refugium for temperate and subtropical species during the cold phases of the Quaternary [10,71]. This region likely provided a refugium for *S. bifidus* during adverse climatic conditions, allowing it to maintain its genetic diversity and population size. Our study’s divergence time estimation suggests that the split between the two major clades of *S. bifidus* occurred around 472 thousand years ago (Ka), during the Middle Pleistocene. This period was marked by alternating glacial and interglacial stages, which might have influenced the species’ distribution and population dynamics.

The Bayesian skyline plot analysis indicated an initial increase in the effective population size of all the samples, and specifically, Clade A, before the third glaciation period of the penultimate glacial [72], while Clade B’s population size increase occurred during this glaciation. This pattern of population expansion aligns with findings from other phylogeographic studies in China, suggesting that several species began expanding their populations before the last glacial maximum, likely emerging from glacial refugia in the southwest [73,74].

The expansion of *S. bifidus* from southwestern to eastern China during the Middle Pleistocene suggests a dynamic response to changing climatic and ecological conditions, with populations extending their range through colonization as conditions became favorable [10]. This expansion is further supported by ecological niche modeling analyses, indicating the suitability of southern coastal China and Hainan Island since the last interglacial period. The formation of land bridges during lower sea levels facilitated the spread of populations to Hainan Island, with a subsequent sea-level rise leading to isolation [5]. A second significant increase in the effective population size of Clade A around 60 Ka, during the Late Pleistocene, may reflect an adaptation to increasing climatic suitability. This period of population expansion underscores the adaptability of *S. bifidus* to changing environments and its ability to exploit new habitats as they become available. 

## 5. Conclusions

This study elucidated the phylogeographic patterns of *S. bifidus* populations in southern China, delineating the species into two distinct clades. A key finding of this study is the identification of the Qiongzhou Strait’s land bridge as a pivotal “dispersal corridor” during the Middle Pleistocene glaciation period. This natural bridge facilitated the movement of *S. bifidus* from Mainland China to Hainan Island, with subsequent sea-level rises leading to the isolation of the Hainan population as the strait became submerged. Furthermore, the expansion of *S. bifidus* during the Late Pleistocene is attributed to favorable climatic conditions that likely supported its rapid spread across its current range. This study underscores the profound influence of Pleistocene sea-level changes and climatic oscillations on the distribution and evolutionary trajectories of *S. bifidus* in China. 

Despite these advancements, challenges such as the limited sampling in specific localities, the long-time duration of sampling, and the constraints of current molecular markers have impeded our ability to precisely resolve the dispersal routes from Mainland China to Hainan Island and more detailed demographic histories, or to explore local adaptations among the various populations of *S. bifidus*. These scientific questions can be further explored by expanding our sample collection, utilizing genome-wide molecular markers more comprehensively, and designing detailed and rigorous experimental protocols. This approach will enhance our understanding of the genetic structure and evolutionary dynamics of *Sycanus bifidus* across its distribution range. By integrating extensive geographic sampling with advanced genomic tools, we can better elucidate the mechanisms driving the diversity and adaptation of this species in various environmental contexts. Nevertheless, the insights gained into the phylogeographic patterns provide a valuable foundation for understanding the evolutionary history of assassin bugs in East Asia. This knowledge is crucial not only for appreciating the biogeographic history of this species, but also for contributing to sustainable agricultural practices and biodiversity conservation efforts.

## Figures and Tables

**Table 1:** Genetic diversity, neutrality test, and statistics of mismatch distribution analyses based on mitochondrial genes.

Code	*N*	*S*	*H*	*Hd*	*π*	Tajima’s *D*	Fu’s *Fs*	*SSD*	*r*
All	65	1028	62	0.998	0.00715	−1.847 **	−9.294 *	0.00630 **	0.00128
Clade A	37	627	35	0.995	0.00477	−2.038 **	−4.078	0.00221	0.00371
Clade B	28	552	27	0.997	0.00445	−2.141 **	−3.075	0.00549	0.00940
Hainan Island	10	181	10	1.000	0.00307	−1.503 *	−0.414	0.01668	0.02568

*N*, number of samples; *S*, segregating sites; *H*, haplotype number; *Hd*, haplotype diversity; *π*, nucleotide diversity; *SSD*, sum of square deviations; *r*, Harpending’s raggedness index. * *p* < 0.05; ** *p* < 0.01.

## Data Availability

The genome resequencing data generated in the present study have been deposited in the GenBank Sequence Read Archive (SRA) database under the BioProject accession number PRJNA1033151. The mitochondrial and nuclear ribosome RNA sequences underlying this study have also been deposited in GenBank with the accession numbers OR734017–OR734077 and OR743562–OR743622. The analytical matrices and resulting tree files have been deposited in the figshare database and are available at: https://figshare.com/s/caeee838431cbf080086 (accessed on 28 February 2024).

## Supplementary Materials

The following supporting information can be downloaded at: https://www.mdpi.com/article/10.3390/biology13050305/s1, Figure S1: Phylogenetic relationships of *Sycanus bifidus* derived from mitochondrial gene sequences; Figure S2: Haplotype network for *S. bifidus* constructed from the concatenated mitochondrial genes; Table S1: Information on the *Sycanus bifidus* specimens used in this study. Table S2: Best-fit schemes and substitution models for phylogenetic analyses; Table S3: Best-fit schemes and substitution models for estimation of divergence time; Table S4: Genetic diversity based on different dataset; Table S5: The environmental variable contribution to the Maxent model in ENM analyses for present, LGM, and LIG periods.
