# Supplementary material for: Phylogeographic Pattern of the Assassin Bug Sycanus bifidus Inferred from Mitochondrial Genomes and Nuclear Genes
Source: Biology (Basel). 2024 Apr 28;13(5):305. doi: 10.3390/biology13050305 (PMC11118239; doi:10.3390/biology13050305)
Supplement: Supplementary file 1 [file biology-13-00305-s001.zip › biology-2974272-supplementary.pdf]

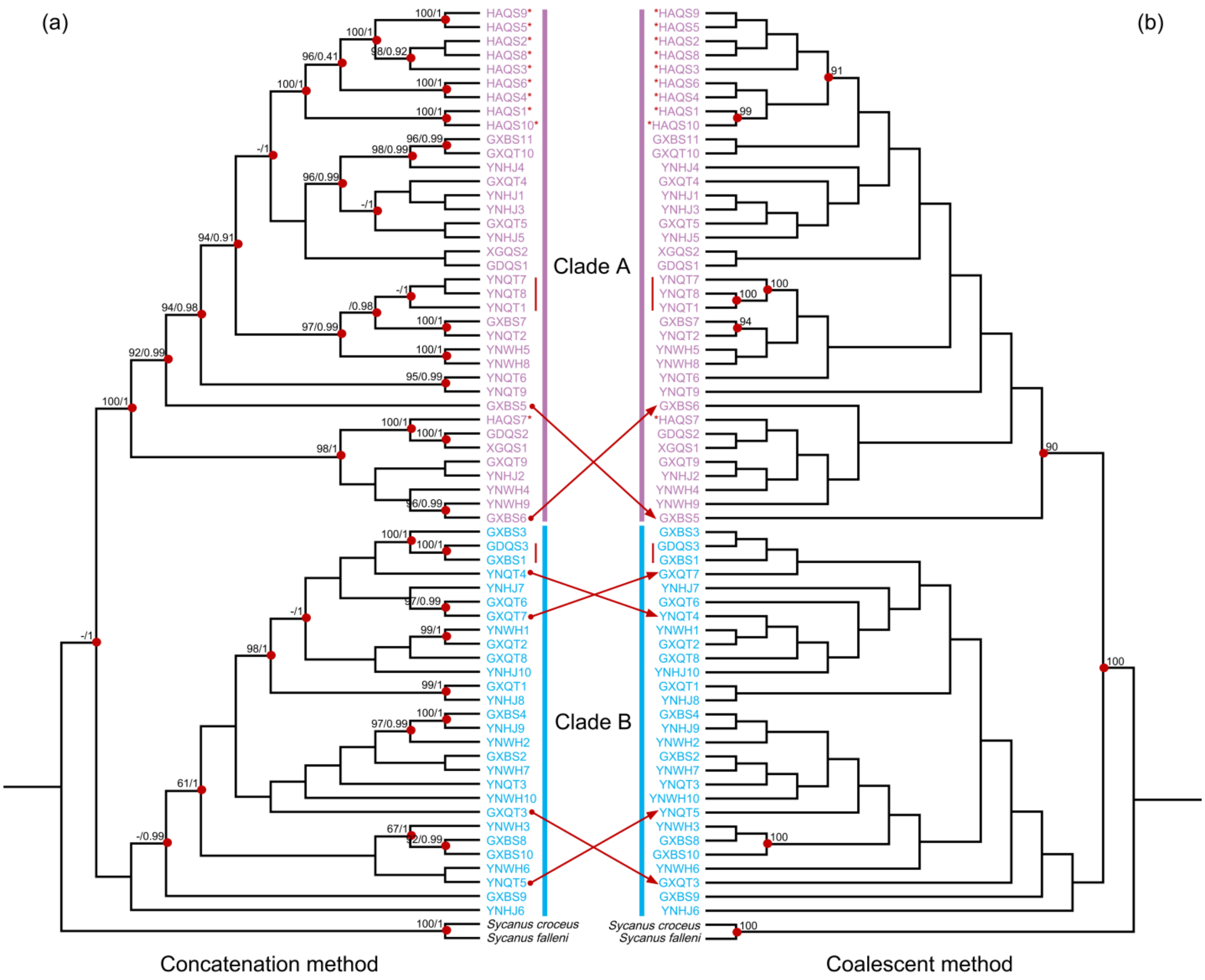

Figure S1. Phylogenetic relationships of *Sycanus bifidus* derived from mitochondrial gene sequences. (a) Tree topology conducted with the concatenation method. The numbers at the nodes represent the bootstrap supports (red nots, > 90) in Maximum-likelihood analysis using IQ-TREE and posterior probability (red nots, > 0.9) in Bayesian inference analysis using MrBayes. (b) Tree topology from multispecies coalescent (MSC) analysis performed with ASTRAL. Numbers at the nodes indicate posterior probabilities of MSC (red nots, > 90). (a, b) Phylogeographic clades are differentiated by colors. Individuals from Hainan Island are denoted with red asterisks, and those sharing the same haplotype are indicated with red lines. Topological discrepancy between concatenation and coalescent methods are indicated with red arrows.

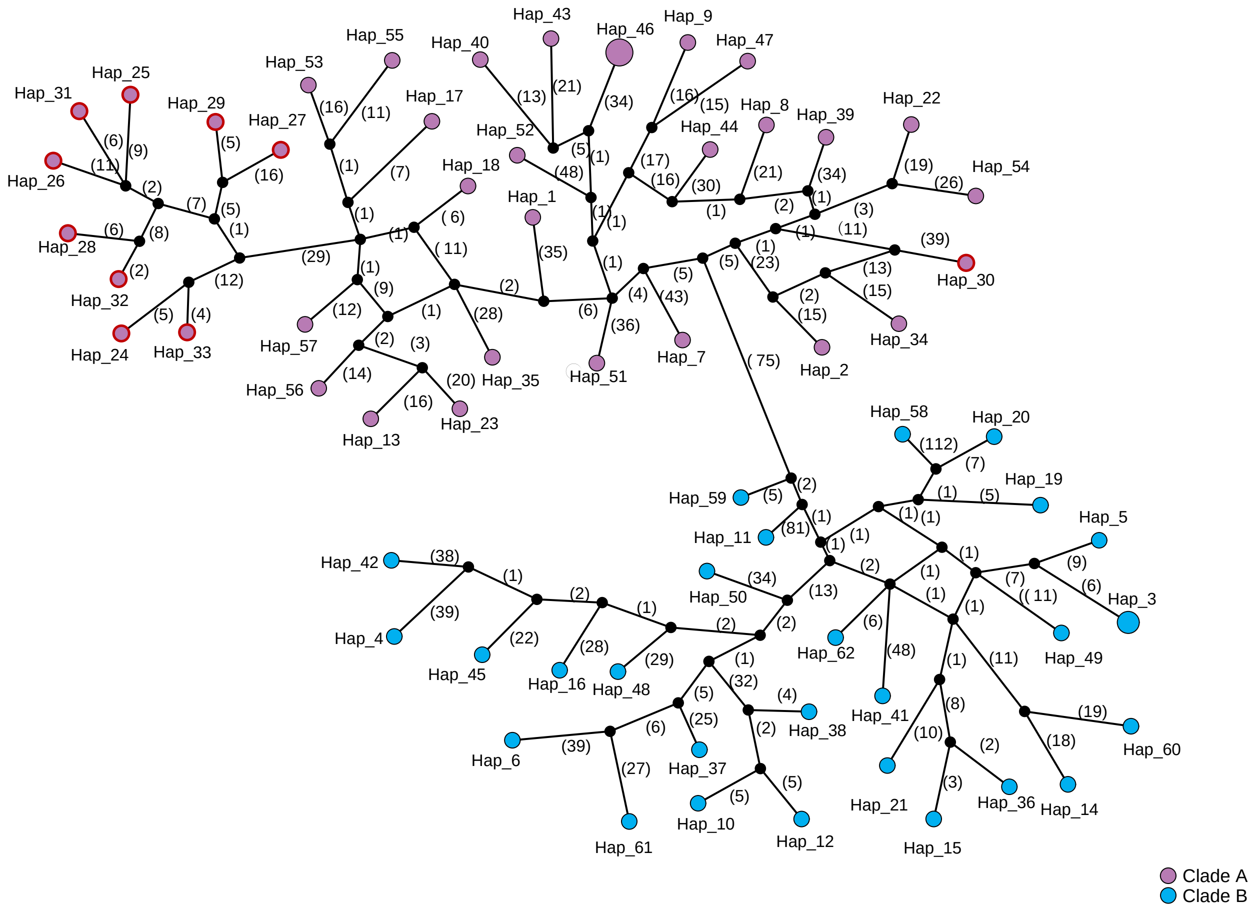

Figure S2. Haplotype network for *S. bifidus* constructed from the concatenated mitochondrial genes. Each colored circle corresponds to a distinct observed haplotype, while black circles indicate hypothesized haplotypes not detected in the samples. Numbers along the connecting lines denote the number of mutation steps between haplotypes. Circle size is proportional to haplotype frequency. The colors of the circles match those used in Figure 3 to denote the same phylogeographic clade. Haplotypes identified in the Hainan Island population are enclosed in a red outline.

Table S1. Information for *Sycanus bifidus* specimens used in this study.

Code	Sample locality	Locality code	Longitude (E)	Latitude (N)	Collection dates	MG	NG	Haplotype code (concatenated dataset)	Haplotype code (mitochondrial genes)
YNWH1	Malipo, Wenshan, Yunnan	1	104.70	23.12	08/17/2015	√	√	Hap16	Hap36
YNWH2	Mt. Huanglian, Lvchun, Yunnan	2	102.40	22.99	05/10/2012	√	√	Hap17	Hap37
YNWH3	Malipo, Wenshan, Yunnan	1	104.70	23.12	08/17/2015	√	√	Hap18	Hap38
YNWH4	Mt. Huanglian, Lvchun, Yunnan	2	102.40	22.99	05/10/2012	√	√	Hap19	Hap39
YNWH5	Mt. Huanglian, Lvchun, Yunnan	2	102.40	22.99	05/10/2012	√	√	Hap20	Hap40
YNWH6	Mt. Huanglian, Lvchun, Yunnan	2	102.40	22.99	05/10/2012	√	√	Hap21	Hap41
YNWH7	Mt. Huanglian, Lvchun, Yunnan	2	102.40	22.99	05/10/2012	√	√	Hap22	Hap42
YNWH8	Mt. Huanglian, Lvchun, Yunnan	2	102.40	22.99	05/10/2012	√	√	Hap23	Hap43
YNWH9	Mt. Huanglian, Lvchun, Yunnan	2	102.40	22.99	05/10/2012	√	√	Hap24	Hap44
YNWH10	Mt. Huanglian, Lvchun, Yunnan	2	102.40	22.99	06/11/2013	√	√	Hap25	Hap45
YNHJ1	Hekou, Honghe, Yunnan	3	103.33	22.53	05/20/2011	√	√	Hap6	Hap53
YNHJ2	Jinping, Honghe, Yunnan	4	103.22	22.78	09/24/2012	√	√	Hap7	Hap54
YNHJ3	Hekou, Honghe, Yunnan	3	103.33	22.53	05/20/2011	√	√	Hap8	Hap55
YNHJ4	Hekou, Honghe, Yunnan	3	103.33	22.53	05/20/2011	√	√	Hap9	Hap56
YNHJ5	Hekou, Honghe, Yunnan	3	103.33	22.53	05/20/2011	√	√	Hap10	Hap57
YNHJ6	Hekou, Honghe, Yunnan	3	103.33	22.53	05/20/2011	√	√	Hap11	Hap58
YNHJ7	Hekou, Honghe, Yunnan	3	103.33	22.53	05/20/2011	√	√	Hap12	Hap59
YNHJ8	Hekou, Honghe, Yunnan	3	103.33	22.53	05/20/2011	√	√	Hap13	Hap60
YNHJ9	Hekou, Honghe, Yunnan	3	103.33	22.53	05/20/2011	√	√	Hap14	Hap61
YNHJ10	Hekou, Honghe, Yunnan	3	103.33	22.53	05/20/2011	√	√	Hap15	Hap62
YNQT1	Mt. Jinuo, Jinghong, Yunnan	9	100.99	22.07	07/25/2021	√	√	Hap1	Hap46
YNQT2	Simao, Puer, Yunnan	6	100.97	22.78	05/13/2018	√	√	Hap2	Hap47
YNQT3	Mengla, Xishuangbanna, Yunnan	10	101.56	21.46	05/08/2015	√	√	Hap3	Hap48
YNQT4	Yuanjiang, Yuxi, Yunnan	5	102.03	23.34	08/18/2014	√		Hap49	Hap49
YNQT5	Mojiang, Puer, Yunnan	7	100.97	22.83	09/05/2005	√		Hap50	Hap50

YNQT6	Fengqing, Lincang, Yunnan	8	100.08	24.58	06/20/2005	√	√	Hap51	Hap51
YNQT7	Mt. Jinuo, Jinghong, Yunnan	9	100.99	22.07	07/25/2021	√	√	Hap1	Hap46
YNQT8	Mt. Jinuo, Jinghong, Yunnan	9	100.99	22.07	07/25/2021	√	√	Hap1	Hap46
YNQT9	Mengla, Xishuangbanna, Yunnan	10	101.56	21.46	03/24/2013	√	√	Hap5	Hap52
GDQS1	Yangchun, Yangjiang, Guangdong	12	111.79	22.17	04/30/2002	√	√	Hap44	Hap1
GDQS2	Huicheng, Huizhou, Guangdong	11	114.38	23.08	08/19/2004	√	√	Hap45	Hap2
GDQS3	Meixian, Meizhou, Guangdong	13	116.10	24.29	09/06/1981	√	√	Hap26	Hap3
GXBS1	Napo, Baise, Guangxi	15	105.84	23.41	08/10/2016	√	√	Hap26	Hap3
GXBS2	Leye, Baise, Guangxi	14	106.56	24.78	08/28/2006	√	√	Hap27	Hap4
GXBS3	Napo, Baise, Guangxi	15	105.84	23.41	08/10/2016	√	√	Hap28	Hap5
GXBS4	Napo, Baise, Guangxi	15	105.84	23.41	08/10/2016	√	√	Hap29	Hap6
GXBS5	Napo, Baise, Guangxi	15	105.84	23.41	08/10/2016	√			Hap7
GXBS6	Napo, Baise, Guangxi	15	105.84	23.41	08/10/2016	√	√	Hap30	Hap8
GXBS7	Napo, Baise, Guangxi	15	105.84	23.41	08/10/2016	√	√	Hap31	Hap9
GXBS8	Napo, Baise, Guangxi	15	105.84	23.41	08/10/2016	√	√	Hap32	Hap10
GXBS9	Napo, Baise, Guangxi	15	105.84	23.41	08/10/2016	√	√	Hap33	Hap11
GXBS10	Napo, Baise, Guangxi	15	105.84	23.41	08/10/2016	√	√	Hap34	Hap12
GXBS11	Napo, Baise, Guangxi	15	105.84	23.41	08/10/2016	√	√	Hap35	Hap13
GXQT1	Longzhou, Chongzuo, Guangxi	19	106.98	22.57	05/26/2020	√	√	Hap36	Hap14
GXQT2	Mt. Daming, Wuming, Guangxi	17	108.28	23.16	05/26/2020	√	√	Hap37	Hap15
GXQT3	Shiwandashan, Shangsi, Guangxi	16	107.98	22.15	05/24/2017	√	√	Hap38	Hap16
GXQT4	Shiwandashan, Shangsi, Guangxi	16	107.98	22.15	05/24/2017	√	√	Hap39	Hap17
GXQT5	Shiwandashan, Shangsi, Guangxi	16	107.98	22.15	05/24/2017	√	√	Hap40	Hap18
GXQT6	Longrui, Ningming, Guangxi	20	107.07	22.14	05/18/2006	√	√	Hap41	Hap19
GXQT7	Fusui, Chongzuo, Guangxi	18	107.90	22.63	08/18/2004	√	√	Hap42	Hap20
GXQT8	Longzhou, Chongzuo, Guangxi	19	106.98	22.57	05/26/2020	√			Hap21
GXQT9	Longzhou, Chongzuo, Guangxi	19	106.98	22.57	05/26/2020	√			Hap22
GXQT10	Shiwandashan, Shangsi, Guangxi	16	107.98	22.15	05/24/2017	√	√	Hap43	Hap23
XGQS1	Lidao, Hong Kong	21	113.92	22.22	05/12/2019	√	√	Hap46	Hap34

XGQS2	Lidao, Hong Kong	21	113.92	22.22	05/22/2019	√	√	Hap47	Hap35
HAQS1	Yinggeling, Baisha, Hainan	22	109.35	18.92	10/08/2008	√	√	Hap48	Hap24
HAQS2	Yinggeling, Baisha, Hainan	22	109.35	18.92	10/08/2008	√	√	Hap49	Hap25
HAQS3	Yinggeling, Baisha, Hainan	22	109.35	18.92	10/08/2008	√	√	Hap50	Hap26
HAQS4	Yinggeling, Baisha, Hainan	22	109.35	18.92	10/08/2008	√			Hap27
HAQS5	Fanyang, Wuzhishan, Hainan	24	109.52	18.78	10/26/2008	√	√	Hap51	Hap28
HAQS6	Yinggeling, Baisha, Hainan	22	109.35	18.92	05/30/2008	√	√	Hap52	Hap29
HAQS7	Yinggeling, Baisha, Hainan	22	109.35	18.92	05/30/2008	√	√	Hap53	Hap30
HAQS8	Yinggeling, Baisha, Hainan	22	109.35	18.92	05/30/2008	√	√	Hap54	Hap31
HAQS9	Jianfengling, Ledong, Hainan	23	108.90	18.73	05/10/2007	√	√	Hap55	Hap32
HAQS10	Yinggeling, Baisha, Hainan	22	109.35	18.92	04/28/2008	√	√	Hap56	Hap33

"√", indicates that mitogenome or nuclear rRNA genes have been obtained.

Table S2. Best-fit schemes and substitution models for phylogenetic analyses.

Subset	Best model	Partition
1	TRN+I	*28SrRNA, 18SrRNA, 5_8SrRNA, cox1_pos1, cox3_pos1, cob_pos1, cox2_pos1, cox2_pos2, cox1_pos2, cob_pos2, cox3_pos2, nad4l_pos2, nad6_pos2, nad5_pos2, atp8_pos2, atp6_pos2, nad2_pos2, nad1_pos2, nad3_pos2, nad4_pos2*
2	TIM+G	*nad4_pos3, nad4l_pos3, nad5_pos3, nad1_pos3, nad2_pos3, cox1_pos3, atp6_pos3, cox3_pos3, nad3_pos3, cob_pos3, cox2_pos3, nad6_pos3, atp8_pos3*
3	TIM+I+G	*16SrRNA, ITS2Misc_RNA, ITS1Misc_RNA, nad1_pos1, nad4l_pos1, nad5_pos1, nad4_pos1, nad6_pos1, nad3_pos1, atp6_pos1, atp8_pos1, 12SrRNA, nad2_pos1, 22tRNA*

Table S3. Best-fit schemes and substitution models for estimation of divergence time.

Subset	Best model	Sites	Partition
1	GTR+I+G+X	11076	*cox1*, 12*PCG*s
2	GTR+I+G+X	2370	2*ITS*s, 22*tRNA*s
3	GTR+I+G+X	8120	5*rRNA*s

Table S4. Genetic diversity based on different dataset.

Dataset	*N*	*S*	*H*	*Hd*	π
Mitogenomic dataset	65	1027	62	0.998	0.00714
Nuclear rRNA dataset	59	4	4	0.192	0.00005
Concatenated dataset	59	970	56	0.998	0.00490

N, samples number; *S*, segregating sites; *H*, haplotype number; *Hd*, haplotype diversity; π, nucleotide diversity.

Table S5. The environmental variable contribution to the Maxent model in ENM analyses for present, LGM and LIG periods.

Variable	Present	LGM	LIG
BIO1	1.7	0.2	0.3
BIO5	6.1	2.4	7.1
BIO6	10.5	9.1	38.4
BIO8	9.4	7.3	5.7
BIO9	0.4	0.5	20.6
BIO10	0	0	0
BIO11	37.1	37.1	9.1
BIO14	2.9	3.6	8.2
BIO17	22.3	24.9	8.8
BIO19	9.5	14.7	1.8
